# Deployment and clearance of microrobots for localized therapy: past, present and future

**DOI:** 10.1093/nsr/nwag272

**Published:** 2026-05-27

**Authors:** Ben Wang, Kai Fung Chan, Philip Wai Yan Chiu, Joseph Jao Yiu Sung, Li Zhang

**Affiliations:** College of Chemistry and Environmental Engineering, Shenzhen University, Shenzhen 518055, China; Chow Yuk Ho Technology Centre for Innovative Medicine, The Chinese University of Hong Kong, Hong Kong 999077, China; Li Ka Shing Institute of Health Sciences, The Chinese University of Hong Kong, Hong Kong 999077, China; Chow Yuk Ho Technology Centre for Innovative Medicine, The Chinese University of Hong Kong, Hong Kong 999077, China; Department of Surgery, Chinese University of Hong Kong, Hong Kong 999077, China; Lee Kong Chian School of Medicine, Nanyang Technological University, Singapore 637371, Singapore; Chow Yuk Ho Technology Centre for Innovative Medicine, The Chinese University of Hong Kong, Hong Kong 999077, China; Department of Surgery, Chinese University of Hong Kong, Hong Kong 999077, China; Department of Mechanical and Automation Engineering, The Chinese University of Hong Kong, Hong Kong 999077, China

**Keywords:** microrobots, localized therapy, biohybrid, magnetic actuation, swarm, degradation

## Abstract

Microrobots hold great promise for active and localized therapy by enabling precise navigation and intervention in areas inaccessible to conventional medical devices. However, clinical translation remains hindered by challenges such as navigating complex environments, achieving precise control under dynamic physiological conditions, and ensuring safe post-therapy clearance. This minireview encompasses recent advancements, including biohybrid microrobots that merge synthetic and biological components to improve biocompatibility and functionality, and catheter-assisted systems that integrate conventional medical tools with robotics for enhanced precision and adaptability. It also discusses strategies to tackle key challenges, such as propulsion mechanisms, closed-loop control, biodegradable designs, focusing on improving safety, reliability, and clinical applicability. By compiling these developments, this work outlines a roadmap toward autonomous, multifunctional microrobotic systems capable of navigating complex physiological environments and delivering targeted therapies with minimal invasiveness.

## Teaser

work together with clinical tools, and be safely cleared from the body after treatment.

## INTRODUCTION

Clinical devices, such as medical catheters, guidewires, and endoscopes, play a longstanding critical role in interventional procedures by navigating through cavities or lumens within the human body [1]. These devices are typically connected to external systems, including imaging modalities (e.g. fluoroscopy or ultrasound), power sources, and control units, which deliver real-time visualization, energy input, and precise manipulation during procedures. This connection ensures stability and control, enabling clinicians to perform complex maneuvers like tissue biopsy, ablation, or stent placement with high reliability and safety. However, conventional interventional tools span markedly different size scales and steering mechanisms, and these features fundamentally constrain their accessible workspace in deep or tortuous anatomy. Clinical guidewires and microcatheters typically have outer diameters ranging from several hundred micrometers to about 1 mm, whereas therapeutic endoscopes are generally millimeter- to centimeter-scale and are therefore restricted to relatively larger natural lumens. Their maneuverability is primarily achieved through proximal translation and torque transmission along a long compliant shaft, meaning that effective distal steering is constrained by shaft stiffness, torsional loss, friction, and anatomical curvature. In highly tortuous or narrow branches, the practical limitation is often not the absence of bendability per se, but the difficulty of maintaining precise distal orientation and advancement without buckling, wall contact, or loss of tip responsiveness [2]. Unlike tethered devices, microrobots do not require external connections because they incorporate their own actuation methods, like magnetic propulsion, and onboard sensors, allowing for autonomous or semi-autonomous operation [3]. While tethered tools offer stability and control during procedures, they are limited in reaching narrow or winding pathways. Microrobots, owing to their small size and untethered operation, may overcome some of these limitations by improving maneuverability and navigation precision in otherwise difficult-to-access environments [2]. Here, maneuverability refers to the ability of a robot to navigate complex and narrow pathways, adapt to dynamic environments, and perform precise actions with minimal disruption to surrounding tissues. This may allow microrobots to access previously difficult-to-access regions, such as capillaries or hypoxic tumor zones, while potentially reducing damage to surrounding tissues under appropriately controlled conditions. Nevertheless, microrobots still face challenges in navigation, control, and safety that they must overcome to match the reliability and effectiveness of traditional devices.

To realize these potential advantages *in vivo*, microrobotic systems must be supported by several tightly integrated components, including microrobotic actuators, external actuation systems, and medical imaging modalities for real-time tracking and localization [4]. For example, magnetic actuation relies on external systems to generate controlled magnetic fields, while microrobots are programmed to respond to these fields through embedded magnetic materials or structures, thereby facilitating precise navigation in dynamic environments [5]. To further enhance the maneuverability and adaptability of microrobots, advanced robotic designs integrating multi-degree-of-freedom actuation systems have been proposed. These systems leverage flexible joints and soft robotics principles to enable dynamic shape adaptation and precise control in complex environments. In selected image-guided navigation studies conducted in confined or branched luminal models, microrobotic or highly miniaturized untethered systems have shown lower endpoint positioning error or improved access to narrow targets than larger conventional devices under the same experimental setup. However, such performance depends strongly on the imaging modality, localization method, flow conditions, and anatomical constraints, and should therefore be interpreted in a context-specific manner [6].

Recent advancements in closed-loop control systems and artificial intelligence (AI)-driven algorithms have further enhanced microrobotic navigation and adaptability [7]. AI-based predictive models optimize navigation paths in real time, enabling microrobots to avoid/penetrate obstacles such as blood-brain or mucosal barriers while maintaining precise trajectories [7,8]. More broadly, dynamic responsiveness of microrobots in complex *in vivo* environments is increasingly being achieved through two complementary routes. One relies on integration with external imaging modalities, such as ultrasound, fluoroscopy, magnetic resonance imaging (MRI), and optical imaging, which provide real-time localization, anatomical context, and feedback for closed-loop navigation within clinically established workflows [9]. The other is emerging from microrobots with intrinsic sensing or self-reporting capabilities, in which onboard or material-encoded functions enable direct readout of local biochemical or physical cues and, in some cases, adaptive behavioral regulation. In parallel, representative recent studies have further demonstrated micro/nano-robotic systems with increasingly sophisticated sensing, decision-making, and programmable transluminal functionality, highlighting a broader shift from simple externally driven motion toward more adaptive, information-aware, and task-responsive microrobotic operation [10–12]. While imaging-assisted strategies remain particularly important for deep-tissue localization, global anatomical guidance, and procedural compatibility, intrinsic sensing approaches may reduce dependence on continuous external tracking and enhance local environmental awareness [13–15]. This capability is particularly important in the vascular and cerebrospinal fluid (CSF) systems, where flow dynamics are complex and highly variable. Despite rapid progress in microrobotics for localized therapy, meaningful comparison across different platforms remains challenging. Reported performances are often obtained under highly heterogeneous experimental conditions, including different propulsion mechanisms, robot dimensions, imaging modalities, biological environments, and therapeutic objectives. As a result, no single microrobot design can currently be regarded as universally optimal for clinical translation. Instead, translational suitability should be evaluated in a context-dependent manner, with particular attention to *in vivo* controllability, compatibility with clinical workflows, therapeutic function, and the safety of post-treatment degradation, retrieval, or clearance.

This minireview focuses on the critical challenges and advancements related to the deployment, navigation, and clearance of microrobots for localized therapy *in vivo*. We primarily focus on untethered microrobotic systems for *in vivo* localized therapy at the microscale, while nanoscale active systems are mentioned only selectively where they provide relevant supplementary context for propulsion, deployment, or post-therapy fate. Rather than implying a uniform developmental trajectory for all microrobotic systems, this minireview highlights the most clinically consequential bottlenecks and discusses them from a translational perspective that prioritizes controllability, integration, and post-therapy safety. Specifically, we discuss innovative propulsion mechanisms that have shown promise for improving navigation and targeted delivery in complex physiological environments. Furthermore, we explore the integration of closed-loop control systems and AI-driven algorithms to address navigation and adaptability challenges, with the aim of improving microrobotic performance in dynamic and unpredictable biological conditions. More importantly, we emphasize strategies to ensure the safe clearance of microrobots post-therapy, including biodegradable materials and bioresponsive designs, which may help reduce potential risks associated with residual microrobots. By summarizing these developments, this minireview provides a translationally focused view of microrobotic technologies for localized therapy and highlights the key bottlenecks that must be addressed before broader clinical implementation. Rather than exhaustively reviewing all propulsion, material, or imaging strategies, we discuss these topics selectively in the context of a continuous translational chain spanning deployment, workflow-compatible navigation and control, therapeutic function, and post-therapy degradation, retrieval, or clearance.

## MICROROBOT DESIGN FOR ENHANCED CLINICAL TRANSLATION

The design of microrobots is a critical aspect that determines their functionality, adaptability, and clinical applicability in complex physiological environments. In this context, clinical applicability should be understood not simply as whether a given design can demonstrate proof-of-concept performance, but whether it can realistically support controllable *in vivo* operation, compatibility with clinically relevant actuation and imaging workflows, reproducible manufacturing, and safe post-treatment degradation, retrieval, or clearance. Biohybrid microrobots that integrate synthetic materials with biological components, such as living cells or biomolecules, are attractive from a translational perspective because they may improve biocompatibility and enable functions that are difficult to reproduce using purely synthetic platforms [16]. Integrating biological functions into microrobotic systems can exploit the self-healing properties of living cells, embedded sensors, and dynamic responses to environmental changes, while using cost-effective and environmentally friendly fuels [16]. At the same time, their clinical applicability also depends on factors such as source standardization, batch-to-batch reproducibility, storage stability, immunological safety, and the predictability of *in vivo* behavior. For example, stem-cell-assembled microrobots have shown promise in preclinical models for neural regeneration and functional recovery in spinal cord injuries [17,18]. In addition to these biohybrid features, propulsion mechanisms inspired by various creatures, such as bacterial flagella, have been developed, leveraging helical rotations to achieve efficient locomotion under the low Reynolds number conditions typical of microscale environments [19]. This approach allows microrobots to overcome the dominance of viscous forces, ensuring effective navigation even in highly constrained physiological spaces [20]. However, efficient locomotion alone does not ensure clinical applicability unless it can be maintained under physiologically relevant flow conditions and integrated with clinically practical control and imaging strategies. Additionally, structural innovations, such as shape-memory materials and pH-responsive degradable scaffolds, allow microrobots to navigate tortuous pathways and perform targeted therapeutic interventions with high precision [21]. Their translational relevance, however, depends not only on responsiveness and precision, but also on whether these materials retain sufficient mechanical robustness during operation and degrade into products that can be safely metabolized, cleared, or otherwise managed *in vivo*. These designs are further complemented by modular assembly approaches, where microrobots are constructed with detachable components (including modularly assembled and swarming robots) designed for specific tasks, such as drug delivery, thrombolysis, or tissue regeneration [22]. Such modularity may enhance functional versatility, but it may also introduce additional challenges related to assembly reliability, manufacturing complexity, fragment retention, and post-treatment safety. The incorporation of magnetic materials has been demonstrated to be particularly advantageous, as it enables precise control via external magnetic fields while ensuring deep tissue penetration and minimal invasiveness [23]. Compared with bioinspired propulsion, magnetic actuation is often more readily aligned with real-time navigation *in vivo* because it enables remote, noncontact control and can be integrated more directly with imaging-guided or feedback-based operation, although this advantage must be balanced against hardware complexity and translational practicality. Nevertheless, from a clinical translation standpoint, these advantages must be balanced against issues including long-term material persistence, the fate of residual magnetic components, and the feasibility of complete retrieval or safe clearance after treatment. However, challenges remain in optimizing the balance between structural complexity and operational simplicity, as overly intricate designs may compromise manufacturability and scalability. Overall, no structural design strategy is inherently optimal for clinical translation; rather, clinical applicability emerges from how material choice, actuation mode, imaging compatibility, manufacturability, and post-treatment fate are jointly balanced within realistic *in vivo* constraints.

## PROPULSION MECHANISMS: ADVANCING MICROROBOTIC NAVIGATION AND CONTROL

Microrobotic propulsion is a cornerstone of their design, enabling precise navigation and functionality across various physiological environments [4]. At the microscale, locomotion differs fundamentally from that in macroscopic systems because it occurs in the low Reynolds number regime, where viscous forces dominate and inertial effects are negligible [24]. In such environments, propulsion mechanisms must overcome time-reversible dynamics, as articulated by Purcell’s ‘Scallop Theorem,’ which points out the necessity of nonreciprocal motion for effective locomotion [25]. Biological systems, such as bacterial flagella and spermatozoa, provide useful inspiration for microrobotic propulsion strategies by employing helical rotations and wave-like deformations to achieve efficient movement [26,27].

Magnetic field integration offers precise control, deep tissue penetration, and biocompatibility, making magnetic actuation a promising approach for propulsion [3]. It also addresses some of the limitations of chemical and physical propulsion methods, such as reliance on environmental reactants or susceptibility to Brownian motion at nanoscale [20]. By manipulating magnetic torque and field gradients, microrobots can achieve controlled rotation, translation, and alignment, enabling targeted navigation within dynamic and confined spaces [3,20]. Furthermore, the scalability and adaptability of magnetic actuation allow microrobots to locomote in different physiological environments, from high-viscosity fluids in the vascular system to complex geometries in the CSF [3]. To further enhance the control precision of magnetically actuated microrobots, advanced field-shaping technologies, such as robot-arm-equipped configurations and integrated magnetic-field/imaging platforms, have been developed [28]. These setups allow for fine-tuned manipulation of magnetic gradients and torque with real-time imaging feedback, enabling coordinated swarm behaviors and multitarget therapeutic interventions in vascular networks.

Despite these advancements, achieving reliable control and integration of magnetic actuation systems in dynamic and complex physiological environments remains challenging. This is where closed-loop control strategies become indispensable, as they enable microrobots to adapt dynamically to environmental variability and achieve precise navigation. Closed-loop control systems rely on real-time feedback from high-resolution imaging modalities, such as MRI, X-ray fluoroscopy, or ultrasound, to continuously monitor microrobot positions and adjust actuation parameters accordingly [29]. For magnetically actuated microrobots, closed-loop control strategies involve the dynamic modulation of magnetic field gradients and torque to compensate for environmental variability, such as pulsatile blood flow and high viscosity [29]. Magnetic fields combined with machine learning models have demonstrated the ability to predict and compensate for changes in environmental factors, enhancing microrobot adaptability in complex biological conditions [30]. Moreover, AI-driven predictive models are emerging as a new component of closed-loop control systems, capable of analyzing minimal datasets to optimize navigation paths and actuation sequences while reducing the risk of human error during therapeutic interventions [7]. These models also facilitate autonomous decision-making, allowing microrobots to adapt dynamically to unpredictable physiological changes. However, the performance of navigation and control strategies should be interpreted cautiously, as many demonstrations are still obtained in simplified or partially biomimetic settings. *In vivo* translation remains constrained by factors such as pulsatile flow, tissue heterogeneity, limited imaging resolution at depth, signal attenuation, physiological motion, and the difficulty of maintaining robust feedback in real time. Therefore, reported navigation precision or targeting efficiency should not be directly generalized across anatomical sites or actuation platforms.

## MICROROBOTS FOR *IN VIVO* APPLICATIONS

**Figure 1.**
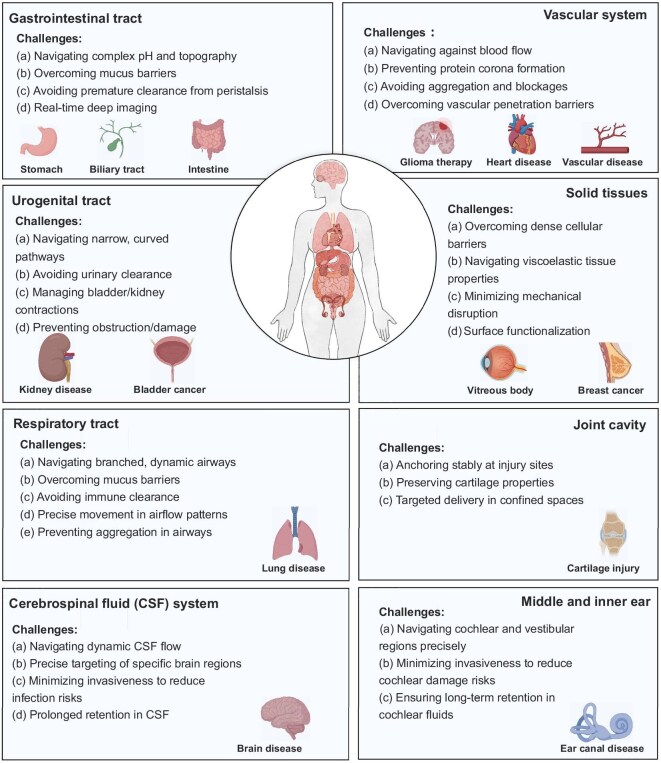
Schematic overview of microrobot applications and challenges across various physiological environments. Insets created in BioRender. He (2026) https://BioRender.com/epvpvt6. The illustration emphasizes the features and obstacles encountered by microrobots in various organs/tissues. Each region has distinct challenges, including navigation through complex structures, overcoming biological barriers, avoiding immune clearance, and achieving precise targeting. Examples of clinical applications are listed for each region, showcasing the potential of microrobots in addressing specific medical needs.

Microrobots offer notable advantages for localized intervention across diverse physiological environments, each with distinct constraints and opportunities. These environments include the gastrointestinal (GI) tract, vascular system, urogenital tract, respiratory tract, joint cavities, solid tissues, and the CSF system, all of which require tailored microrobot designs. Figure 1 provides a schematic overview of these physiological environments and their associated barriers, while Table S1 summarizes representative application scenarios, working mechanisms, and stages of development.

In the GI tract, microrobots encounter challenges such as corrosive digestive fluids and mechanical resistance from peristalsis (the rhythmic contraction of intestinal muscles). These challenges can be mitigated through strategies like chemical propulsion via gastric acid reactions [31] or protective coatings for targeted drug delivery [32]. Capsule-enclosed microrobots and anchored systems augment stability and localization, allowing precise therapeutic interventions at specific sites [33]. In the vascular system, microrobots must navigate high viscosity, rapid blood flow, and pulsatile forces. Strategies such as mimicking neutrophil rolling along vessel walls [34] and leveraging external fields [35] enable targeted drug delivery to distant tissues, with applications including thrombolysis [36] and glioblastoma treatment [37]. Similarly, in the urogenital tract, dynamic electrolyte-rich fluids support self-propelled microrobots powered by environmental substances or magnetically guided systems for precision therapies, such as bladder cancer treatment using oncolytic adenoviruses [38,39]. Within the respiratory tract, microrobots must adapt to the complex, branched, and dynamic airway structure, navigate gas-liquid interfaces, and overcome challenges such as mucus barriers, variable airflow patterns, and immune responses. To address these obstacles, microrobots demonstrate their ability to deliver drugs directly to lung tissues, showing promising efficacy in treating diseases such as bacterial pneumonia [40] and lung cancer [41]. In joint cavities, microrobots overcome challenges posed by viscous synovial fluid and dynamic joint movements through magnetic anchoring systems, enhancing cartilage regeneration at injury sites [42]. In the CSF system, microrobots overcome barriers such as the blood-brain barrier and rapid clearance delivered via bloodstream by directly acting on the CSF. This approach utilizes the relatively slow flow of CSF (0.3–1 cm/s) to achieve precise and controlled targeted delivery, allowing prolonged retention times and locally high drug concentrations. For instance, autologous blood hydrogel fiberbots have been shown to suppress intracranial tumor growth with minimal inflammatory response, while stem-cell-assembled microrobots facilitate neural regeneration and functional recovery in spinal cord injury models by releasing therapeutic cells [17,18,43]. Even in solid tissues, microrobots can navigate in the dense cellular matrices and polymer networks by employing specialized shapes, surface treatments, and propulsion mechanisms. These designs enable precise drug delivery to hypoxic tumor regions [44] or the vitreous body of the eye [45] without obvious tissue damage, demonstrating their potential for minimally invasive therapies. Across these applications, microrobots may improve localization, prolong local retention, and enhance therapeutic efficacy in physiologically challenging environments. Robotic path-planning algorithms have been adapted for microrobots to optimize navigation in physiological environments. These algorithms enable real-time decision-making, allowing microrobots to avoid obstacles like mucosal barriers while maintaining efficient trajectories toward target sites [46].

The long-distance delivery of microrobots is crucial for minimally invasive treatment of deep-seated lesions within the human body. By accessing target sites through natural cavities or via injection and puncture, microrobots reduce the need for direct interventions in sensitive or hard-to-reach areas, minimizing tissue damage, infection risks, and patient discomfort. In the digestive system, spanning several meters and characterized by barriers like stomach acidity, mucosal layers, and intestinal peristalsis, microrobots can be administered orally or rectally to reach specific sites. This avoids invasive procedures or local injections that may cause discomfort or complications. In the circulatory system, microrobots can be introduced into peripheral vessels, such as those in the arm or leg, leveraging natural blood flow to transport them to diseased areas like tumors or inflamed tissues. This reduces risks associated with direct or localized injections near critical regions like the brain or heart. Similarly, in the CSF system, microrobots can be introduced at distal sites, such as the lumbar region, and actively navigate through the spinal canal to deliver drugs precisely to target areas in the brain or central nervous system, improving therapeutic efficacy without direct intervention near sensitive structures.

Despite these advantages, microrobots face notable challenges in navigating the extensive and dynamic pathways of the digestive, circulatory, and CSF systems. The vast scale and complexity of these systems make the delivery process time-consuming and technically challenging. To address these challenges, researchers have developed new strategies for long-distance microrobot delivery. In the vascular system, blood circulation is utilized to transport microrobots over long distances, while external fields are applied to achieve high-dose accumulation at local disease sites [47]. In the digestive system, intestinal peristalsis serves as a natural transport mechanism. Upon reaching target sites, microrobots can be captured by intestinal villi, which selectively trap microrobots with specific sizes and shapes [48]. Additionally, anchoring structures can be designed on microrobot surfaces to enable prolonged retention and localized drug release at disease sites [49]. These approaches necessitate exceptional expertise to navigate the dynamic and ever-changing internal environments, such as fluid flow and peristalsis, ensuring precise and effective delivery.

## DEPLOYMENT OF THE MICROROBOTS USING CLINICAL TOOLS

**Figure 2.**
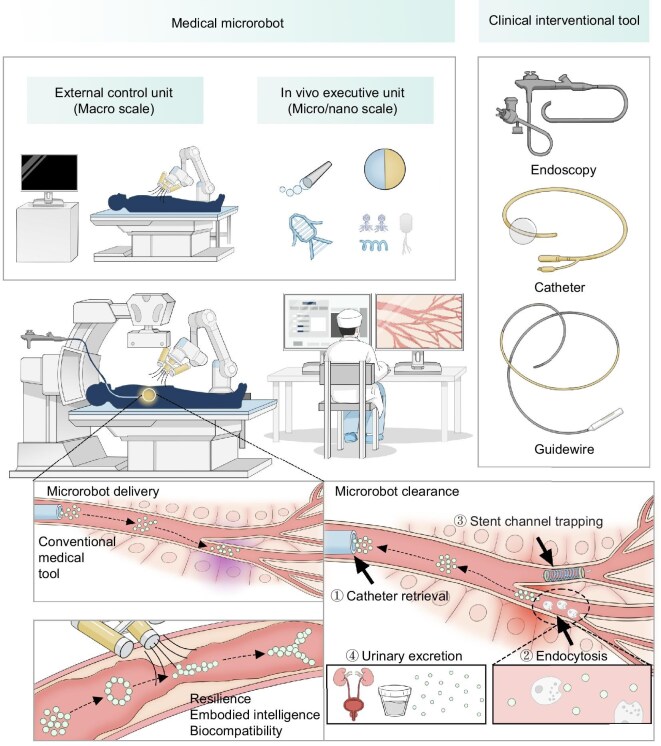
Schematic showing the synergy of conventional medical tools and microrobots for high-precision delivery toward hard-to-access regions inside the body, and the subsequent clearance of the microrobots.

Tethered robots, characterized by their larger size and direct connection to external systems, excel in tasks requiring significant power, precise manipulation, and continuous data transmission. Recent advancements in magnetic catheter/guidewire/endoscopy robots have further expanded the capabilities of interventional surgery, particularly in navigating complex and tortuous lumens with micron-level precision [50,51]. Magnetic navigation systems, which utilize externally applied magnetic fields to exert controlled forces and torques on magnetized devices, enable contactless manipulation and precise spatial reorientation [52–58]. However, their macro-scale design limits their ability to access narrow, tortuous pathways deep within the body, such as sub-millimeter branches of the biliary tract or intricate vascular networks. Conversely, untethered microrobots, defined by their diminutive size and autonomous operation, offer notable maneuverability in confined spaces inaccessible to tethered devices. These microrobots can navigate complex and dynamic physiological environments to perform targeted drug delivery, microsurgery, or localized diagnostics. Accordingly, one important trend in *in vivo* systems is the synergy between traditional medical tools and microrobots. As illustrated in Fig. 2, this concept involves using conventional clinical platforms to assist microrobot deployment into hard-to-access regions, followed by localized action and eventual post-therapy clearance. This synergy aims to extend access to regions that conventional devices cannot readily reach or visualize, thereby enabling more controlled intervention. This approach expands the applicability of existing medical robots. Although the integration of microrobots with established clinical tools may provide a pragmatic translational route, such hybrid systems also introduce new layers of complexity, including coordination between robotic actuation and procedural workflows, device compatibility, imaging-registration accuracy, and the maintenance of stable control under clinically realistic constraints.

**Figure 3.**
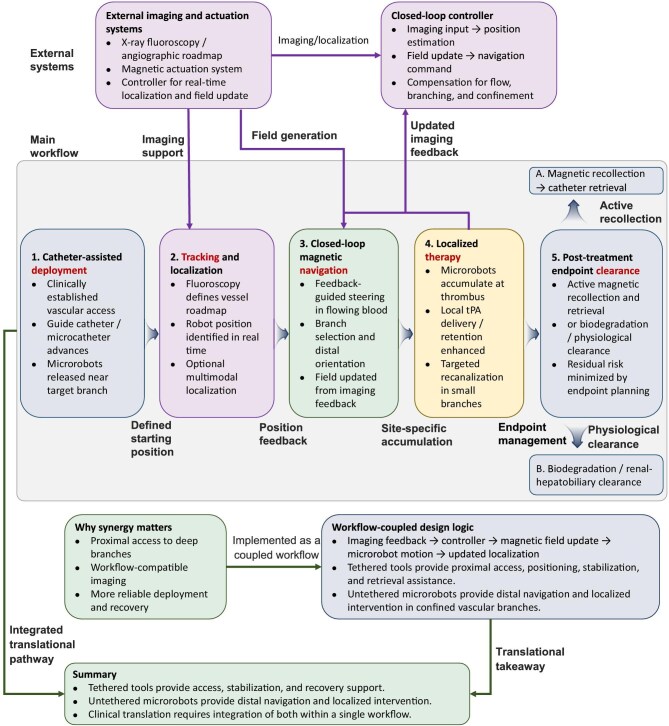
Representative workflow-coupled translational pathway for vascular microrobot deployment, closed-loop navigation, localized therapy, and post-treatment clearance/retrieval.

To make this workflow more clinically explicit, a representative vascular use case is summarized in Fig. 3. In such a workflow, catheter- or microcatheter-assisted access to a major vessel can be used to introduce microrobots closer to the target branch, thereby reducing the need for long-distance untethered traversal from the entry site. In this setting, the tethered device serves as a clinically familiar platform for access, positioning, and deployment, while decreasing the downstream navigation burden placed on the microrobot. After release, the microrobot may be tracked using fluoroscopy, ultrasound, magnetic localization, or multimodal image fusion, depending on anatomical depth, temporal resolution, and compatibility with the actuation field. These tracking signals can then be incorporated into a closed-loop navigation and control loop, allowing the microrobot to adapt its orientation and propulsion in response to branching geometry, blood flow, and local confinement. At the target site, the microrobot may perform localized drug delivery, thrombus interaction, micro-occlusion targeting, or other focal therapeutic actions depending on the intended clinical task. Following therapy, endpoint management becomes an integral part of the workflow rather than a separate consideration, and may involve active retrieval when feasible or biodegradation and physiological clearance depending on the material platform and treatment context.

However, synergizing traditional medical tools with microrobots still presents several challenges, such as the coordination of their hardware and software systems and the management of low signal-to-noise ratios for real-time imaging. These issues align with the primary challenges highlighted in *The Grand Challenges of Science Robotics* [46] within the medical robotics domain. Recent synergized platforms have demonstrated access to fine branches of the digestive system, ear canal, and small blood vessels for minimally invasive localized intervention. This hybrid approach expands the range of clinical situations in which medical robots may be applied, with the potential to enhance their efficacy and precision in medical interventions. For example, endoscopy-assisted magnetic navigation platforms have been developed to transport microrobots over long distances and deploy them in narrow lumens, such as the bile duct or small vascular branches, where conventional tools cannot operate effectively. Dual imaging modalities that combine endoscopic and ultrasound imaging have demonstrated notable potential in enhancing the localization and tracking of microrobots during therapeutic interventions [59]. Similarly, a modular microrobot system designed for targeted cell delivery to the bile duct integrates technical modules, including magnetic actuation and cell scaffold components fabricated using 3D printing technology. The magnetic actuation module features strong magnetic responsiveness and pH-triggered deformability, while the cell scaffold module offers excellent biodegradability for cell loading and release. In the acidic bile duct environment, the microrobot’s pH-responsive contraction triggers disassembly, allowing localized cell delivery. The magnetic module is then retrieved via a catheter, minimizing safety risks [22,60].

The blood circulation system shows another challenging environment due to its tortuous network of decreasing branches. Microrobots offer a highly precise strategy for intravascular therapy, addressing challenges posed by submillimeter segments and the need for intervention in deep tissues [61–65]. For example, retrievable tPA-anchored Fe_3_O_4_@mSiO_2_ nanorobots (tPA-nbots) utilize balloon catheter-assisted magnetic actuation and X-ray fluoroscopy for precise thrombolysis and retrieval [61]. This innovation improves thrombolysis accuracy while enhancing patient safety by enabling effective post-treatment retrieval, thereby reducing potential complications from residual nonbiodegradable materials. Another example is a marsupial robotic system combining chemical/magnetic hybrid nanorobots with a miniature magnetic continuum robot, which has shown promise for intracranial cross-scale drug delivery in glioblastoma treatment [66]. This system navigates through minimally invasive cranial channels to bypass the blood-brain barrier, enabling precise microscale drug targeting and reducing collateral damage to healthy tissue.

Innovations in catheter-assisted treatment for vascular embolization further demonstrate the versatility and effectiveness of these technologies. For aneurysm embolization, microrobots containing pH-responsive self-healing hydrogels and magnetic nanoparticles are deployed and navigated to target sites under imaging guidance, achieving high filling efficiency and long-term stability [67]. The development of untethered shape-memory magnetic microrobots [68,69] and soft adhesive magnetic millirobots [70] showcases advanced capabilities in navigating complex vascular systems and delivering targeted therapies, with applications validated in both *ex vivo* and *in vivo* models.

Despite the promise of synergized systems, several technical challenges remain. Coordinating the hardware and software of tethered and untethered systems is a complex task, requiring seamless communication between imaging, actuation, and microrobot control modules. For instance, real-time imaging of microrobots in deep tissues often suffers from low signal-to-noise ratios, complicating precise navigation and task execution. Additionally, ensuring biocompatibility and minimizing the risk of adverse interactions between microrobots and conventional tools are critical for clinical safety [71]. Developing robust algorithms for autonomous navigation and adaptive control in dynamic environments is essential to overcome these challenges.

## POST-THERAPY CLEARANCE OF THE MICROROBOTS *IN VIVO*

The safe and efficient clearance of untethered microrobots post-therapy is a critical yet underexplored aspect of their clinical translation. The presence of residual microrobots in the body shows long-term biocompatibility risks, particularly for those containing magnetic components that degrade slowly. To address these concerns, clearance strategies must be designed for specific physiological environments and applications, with careful consideration of both therapeutic utility and post-treatment safety. At present, however, the field still lacks standardized quantitative benchmarks for what constitutes clinically acceptable clearance efficiency. In practice, an acceptable residual burden is unlikely to be defined by a universal percentage alone, because its clinical significance depends strongly on the anatomical site, the material composition, the size and number of retained components, their spatial distribution, and the expected duration of retention. For example, a low residual fraction may still be unacceptable if particles are retained in the cerebral circulation, endothelium-sensitive vascular territories, or other functionally critical sites, whereas a somewhat higher transient residual burden may be more manageable in accessible luminal environments if reliable follow-up degradation, capture, or elimination can be demonstrated. Accordingly, future translational studies should report clearance not only as a percentage of removed or degraded material, but also in terms of residual mass or particle number, retention location, clearance half-time, and the proportion of material remaining at clinically relevant time points. New clearance mechanisms have been developed to tackle these challenges. Self-assembling microrobots that disassemble in response to external stimuli, such as magnetic fields or pH changes, enable aggregation into retrievable swarms for simplified removal from sensitive regions. Biodegradable materials, such as magnesium alloys or hydrogel-based composites, further minimize residual accumulation and immune responses, enhancing biosafety. These materials degrade naturally within the body, reducing the need for invasive retrieval procedures. Importantly, biodegradability should not be assumed to be synonymous with safety. Even when a microrobot is designed to disassemble or degrade after therapy, its translational suitability still depends on the chemical identity of degradation products, their local and systemic distribution, the timescale of clearance, potential immune responses, and the reproducibility of these processes *in vivo*. Accordingly, future studies should evaluate not only functional degradation or disappearance of the original structure, but also the biological fate and safety profile of all resulting components under clinically relevant conditions. Importantly, post-therapy fate is highly scale-dependent; therefore, clearance routes applicable to nanoparticles or nanoscale fragments should not be assumed to apply to intact microscale robotic systems. From a translational perspective, the most informative future benchmark may therefore be a context-specific clearance profile rather than a single cutoff value, for example demonstrating near-complete removal or degradation in high-risk sites, together with evidence that any residual fraction does not produce progressive inflammation, embolic obstruction, endothelial injury, neurotoxicity, or other delayed pathology over a predefined observation window.

Active clearance methods, such as catheter retrieval guided by external magnetic fields, offer precise control in vascular systems. This approach enables microrobots to be directed to accessible locations for removal using interventional tools, such as catheters, making it particularly effective in luminal applications where precision is crucial. Nevertheless, retrieval-based approaches may be more practical in anatomically accessible or lumen-confined settings, whereas their applicability may be limited in diffusely distributed, deeply embedded, or poorly visualized targets. For microrobots deployed near stented areas, magnetic stent-channel trapping offers a specialized solution, directing microrobots into stent channels for safe and controlled removal without additional invasive procedures. Similarly, in gastrointestinal applications, magnetic retrieval systems have demonstrated potential for capturing and retrieving microrobots, offering practical solutions for ensuring their safe removal post-therapy. Passive renal clearance is generally relevant not to intact microrobots, but to sufficiently small nanoscale degradation products or disassembled components, whose biological elimination may occur through urinary excretion depending on their hydrodynamic size, composition, and surface properties. Another biologically-driven clearance mechanism involves endocytosis, where smaller microrobots are internalized by cells and subsequently degraded and metabolized by the body’s natural processes. These passive and active strategies collectively may help reduce the risks associated with the long-term presence of microrobots within the body. For nondegradable or slowly degradable magnetic materials, however, retrieval efficiency alone should not be taken as a sufficient safety endpoint. Even a small retained fraction may remain biologically relevant if it persists at sites exposed to high shear stress, endothelial contact, or limited tissue tolerance. In vascular settings, prolonged retention may contribute to chronic endothelial irritation, local inflammatory activation, foreign-body responses, thrombogenicity, disturbed microcirculatory flow, or fibrotic encapsulation depending on particle size, shape, surface chemistry, and retention site. In the central nervous system or CSF pathways, additional concerns include persistent neuroinflammation, microglial activation, interference with local tissue homeostasis, or mechanical obstruction in confined spaces. Therefore, assessment of post-therapy safety for magnetic microrobots should ideally extend beyond retrieval success and include longer-term histopathological and toxicological evaluation, such as endothelial integrity, inflammatory-cell infiltration, thrombosis, gliosis or microglial response where relevant, organ-level biodistribution, and persistence of residual magnetic material over time. Such evaluation is particularly important because current evidence in the microrobotics literature often demonstrates short-term procedural feasibility more clearly than long-term pathological innocuity under clinically realistic retention scenarios.

Designing microrobots with both therapeutic functionalities and clearance strategies is crucial to ensure their safety and efficacy for long-term use. By incorporating multifunctional materials and adaptive clearance mechanisms, microrobots can be seamlessly integrated into clinical workflows, bridging the gap between therapeutic benefits and post-therapy safety. In this regard, clinically meaningful clearance should be understood not simply as whether the original robot structure disappears or is partially retrieved, but whether the entire post-therapy residual burden—including intact bodies, fragments, degradation products, and retained magnetic constituents—falls below a biologically tolerable threshold for the intended anatomical context. Establishing such thresholds will likely require closer integration of robotic performance studies with pathology, toxicology, biodistribution analysis, and longitudinal *in vivo* follow-up. Such an integrated approach not only supports improved biosafety assessment but also may facilitate broader clinical adoption of microrobotic technologies in clinical practice.

## CONCLUSION AND OUTLOOK

**Figure 4.**
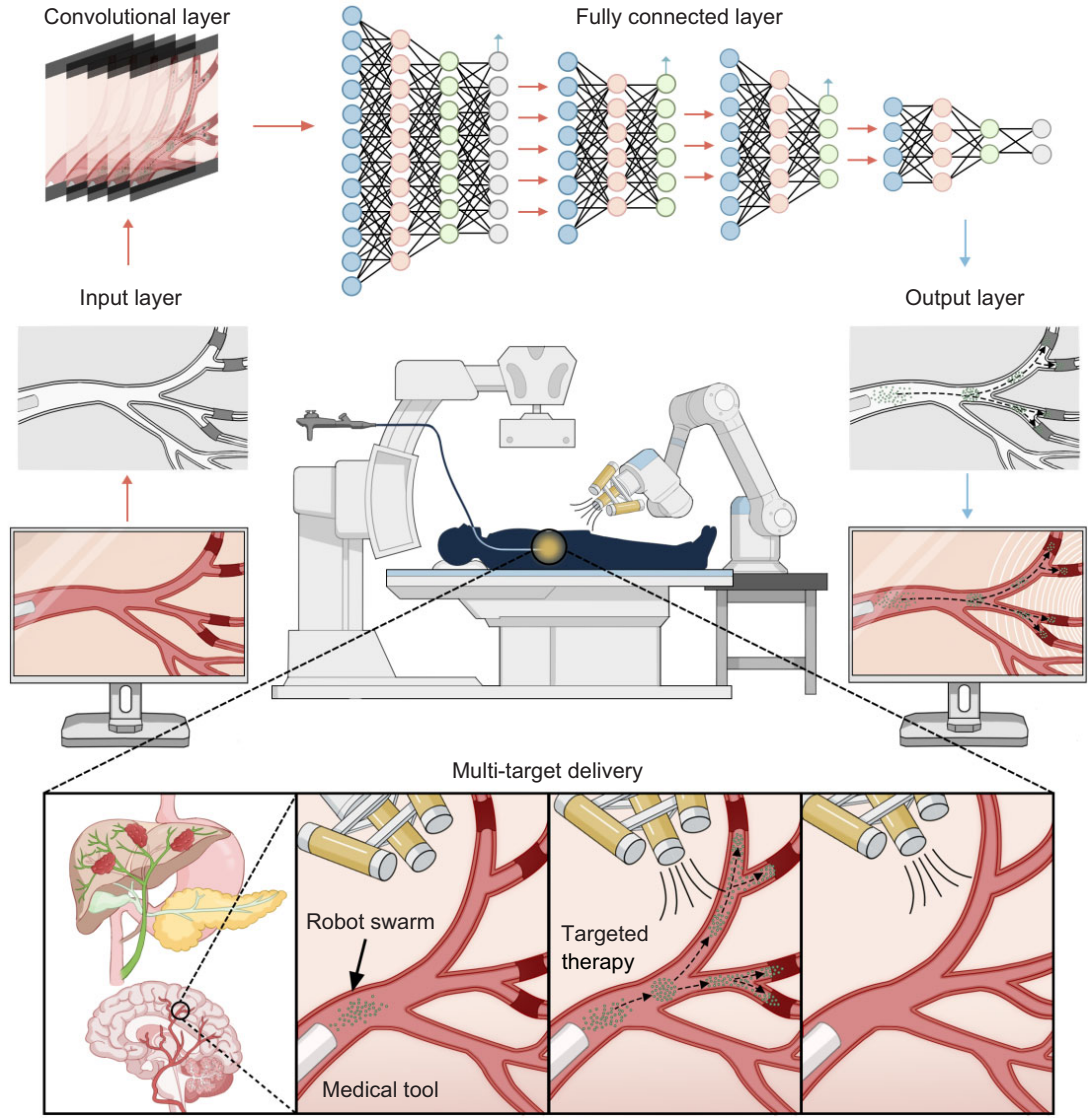
Conceptual outlook of future microrobotic systems by integrating deep learning with robotic magnetic control systems for multitarget coordinated delivery.

Although microrobots have great potential as a precision medicine tool for localized therapy, obstacles including navigation, control, and clearance still stand in the way. This minireview aims to clarify several persistent challenges in the clinical translation of microrobots. Unlike traditional review papers that broadly summarize the field, our work focuses on three critical and underexplored aspects: real-time navigation and control, integration with existing clinical tools, and safe post-therapy clearance. By highlighting these areas, we aim to offer practical insights and future research directions that may facilitate their clinical translation in precision medicine. Looking forward, the most urgent priority for clinical translation may not be increasing the structural or functional complexity of microrobots alone, but establishing reliable performance under realistic physiological conditions. In particular, robust *in vivo* localization and control, compatibility with clinically available deployment and imaging tools, and a credible post-therapy degradation, retrieval, or clearance route should be regarded as foundational translational criteria. Without these elements, even highly sophisticated microrobotic designs may remain difficult to prioritize for clinical use. Ongoing research should further optimize *in vivo* control, improve functional reliability, and ensure safe post-therapy clearance. By addressing these challenges through innovative design and material choices, the synergy of tethered and untethered microrobots can offer safe, efficient, and highly precise solutions for various medical applications, ranging from diagnostics to targeted therapies. Future advancements in microrobotics are expected to further improve their safety and efficacy, supporting their broader use in minimally invasive medicine. Moreover, as conceptually summarized in Fig. 4, future microrobotic delivery systems may increasingly incorporate intelligent control architectures, potentially including deep learning algorithms and fluid dynamics simulation tools, to support more adaptive decision-making, navigation, and task execution. Such systems may support more autonomous navigation and targeted delivery under appropriate clinical supervision. Current efforts are increasingly directed toward data-efficient control models that can adapt to dynamic physiological environments and integrate learning-based prediction with physics-informed guidance. This direction may help reduce operator burden and improve control consistency during complex interventions. Close collaboration with clinicians will be essential for translating these technologies into practice. By working alongside clinicians, researchers can better understand the practical challenges faced in surgical and diagnostic settings, ensuring that microrobots are designed with real-world medical applications in mind. Moreover, this partnership allows for a continuous feedback loop between engineering breakthroughs and clinical practice, enabling more personalized and effective treatment approaches tailored to patient needs. As autonomous functions expand, issues such as safety constraints, interpretability, and appropriate clinician oversight will become increasingly important. As also summarized in Fig. 4, another notable development trend is the transition from single-target intervention to multitarget coordinated delivery, particularly in branched luminal systems where multiple lesions may need to be addressed within a single procedural framework. By shifting towards a multitarget approach, medical robots can enhance their therapeutic efficacy and adaptability, providing more comprehensive treatment solutions for intricate medical conditions. In specific *in vivo* disease scenarios, such as those within the human circulatory and digestive systems where numerous branching lumens exist, small branch lumens may develop lesions at multiple sites. For example, during thrombectomy in large vessels, thrombus fragments may break off and travel downstream, potentially causing embolism in multiple smaller vessels. Similarly, within the digestive system, a tumor occupying a segment of the biliary tract can extend into multiple subsidiary ducts or vessels, complicating the treatment process. As a result, microrobots must be capable of effectively covering Y-shaped regions and navigating into multiple branch lumens to perform targeted tasks. Each microrobot or microswarm can be autonomously directed into different lumens, navigating independently through the complex lumen networks. Once within these lumens, they can execute distinct tasks, such as targeted thrombolysis, drug delivery, and embolism. This approach not only enhances precision in treating multiple lesions simultaneously but also substantially improves overall therapeutic efficacy by enabling localized treatment at each affected lumen, minimizing systemic side effects, and reducing the treatment time compared to conventional single-target therapies. Here, the multiple microrobots or microswarms, once delivered via a catheter, can be controlled externally to autonomously divide into multiple components, each entering different sub-channels within the tumor region to perform multisite therapy. Unlike traditional single-target treatments, this multitarget therapeutic approach offers enhanced treatment efficiency, comprehensive coverage, improved outcomes, reduced side effects and shorter treatment cycles in clinical settings.

It successfully addresses the limitations of single-target therapies, including incomplete treatment, prolonged therapy durations, and significant adverse effects, thereby offering a more robust and comprehensive approach for complicated medical conditions.

## Supplementary Material

nwag272_Supplemental_File
