# Supplementary material for: Deployment and clearance of microrobots for localized therapy: past, present and future
Source: Natl Sci Rev. 2026 May 27;13(12):nwag272. doi: 10.1093/nsr/nwag272 (PMC13309930; doi:10.1093/nsr/nwag272)
Supplement: nwag272_Supplemental_File [file nwag272_supplemental_file.pdf]

Supplementary Materials for

Deployment and clearance of microrobots for localized therapy: past, present and future

Ben Wang[1,†], Kai Fung Chan[3,4,†], Philip Wai Yan Chiu[3,5], Joseph Jao Yiu Sung[6], Li Zhang[2,3,5*]

[1]College of Chemistry and Environmental Engineering, Shenzhen University, Shenzhen 518055, China

[2]Department of Mechanical and Automation Engineering, The Chinese University of Hong Kong, Shatin, New Territories, Hong Kong SAR, China.

[3]Chow Yuk Ho Technology Centre for Innovative Medicine, The Chinese University of Hong Kong, Shatin, New Territories, Hong Kong SAR, China.

[4]Li Ka Shing Institute of Health Sciences, The Chinese University of Hong Kong, Shatin, New Territories, Hong Kong SAR, China

[5]Department of Surgery, Chinese University of Hong Kong, Hong Kong SAR, China

[6]Lee Kong Chian School of Medicine, Nanyang Technological University, Singapore, Singapore

*Corresponding author. Email: lizhang@cuhk.edu.hk (L.Z.)

†These authors contributed equally

This PDF file includes:

Supplementary Table S1

Table S1. Features of clinical methods and microrobotics for targeted delivery.

Body region	Conventional delivery methods	Challenges and limitations	Microrobot design and mechanisms	Functions and applications	Advantages of micro/nanorobot delivery	Stage of Development	References
**Gastrointestinal tract**	- Oral administration (e.g., tablets, capsules).<br>- Endoscopic delivery for localized treatment.<br>- Systemic delivery via bloodstream.	- Low bioavailability due to enzymatic degradation and pH changes.<br>- Limited targeting precision.<br>- Invasive endoscopic procedures.	- Magnetic robots: controlled by external magnetic fields.<br>- Ultrasound-driven robots: powered by acoustic waves.<br>- Autonomous robots: guided by chemical gradients or environmental stimuli.	- Targeted drug delivery to specific GI regions (e.g., duodenum).<br>- Tissue sampling and diagnostics.	- Non-invasive delivery, reducing patient discomfort.<br>- Localized high-concentration drug delivery, minimizing systemic side effects.<br>- Stability in dynamic environments (e.g., peristalsis, gastric acid).	Preclinical (animal models)	[27],[41]
**Vascular system**	- Intravenous injection for systemic drug delivery.<br>- Catheter-based interventional therapies for localized delivery.	- Non-specific drug distribution causing systemic side effects.<br>- Invasive catheter insertion.<br>- Hemodynamic forces disrupting targeting.	- Magnetic robots: navigated by external magnetic fields.<br>- Chemically driven robots: propelled by chemical reactions.	- Targeted delivery to specific organs (e.g., heart, brain, tumors).<br>- Thrombolysis or embolism.	- High-precision targeting, avoiding systemic side effects.<br>- Stable navigation in complex blood flow environments, including the ability to move against blood flow.<br>- Non-invasive operation, reducing surgical risks.	Preclinical (animal models)	[55]
**Urogenital system**	- Systemic delivery via oral or intravenous	- Short retention time due to urinary flow.<br>- Limited precision in	- Magnetic robots: controlled by external magnetic	- Drug delivery to specific	- Non-invasive or minimally invasive delivery, reducing infection risks.	Preclinical (anim	[31],[32],[33]

	administration.<br>- Localized delivery via catheters or stents.	targeting specific tissues.<br>- Invasive catheter procedures with infection risks.	fields.<br>- Ultrasound-driven robots: guided and positioned by acoustic waves.	regions (e.g., bladder, kidneys, reproductive organs).<br>- Localized treatment of urogenital diseases (e.g., bladder cancer).	- Long-term positioning in urinary flow environments.<br>- Stability in high-flow conditions.	al model s)	
**Respir atory system**	- Inhalation therapies (e.g., aerosols, nebulizers).<br>- Systemic drug delivery for lung diseases.	- Difficulty in penetrating deep lung regions.<br>- Uneven drug distribution.<br>- Mucociliary clearance rapidly removes drugs.	- Autonomous robots: actuated by chemical fuel<br>- Magnetic robots: controlled by external magnetic fields.	- Drug delivery to deep lung regions.<br>- Localized treatment of lung diseases (e.g., pneumonia, lung cancer).	- Ability to penetrate mucus barriers and target specific lung regions.<br>- Adaptability to airflow changes, enabling precise navigation.<br>- Non-invasive, reducing patient discomfort.	Precli nical (anim al model s)	[34], [35]
**Solid tissues**	- Systemic drug delivery (e.g., chemotherapy).<br>- Localized delivery via direct injection or implants.	- Difficulty in penetrating dense tissues.<br>- Non-specific targeting may damage surrounding healthy tissues.<br>- Limited drug diffusion in tissues.	- Magnetic robots: capable of penetrating tissues via external magnetic fields.<br>- Chemically driven robots: propelled by chemical reactions within tissues.	- Targeted drug delivery to deep tissues (e.g., tumors or eyes).<br>- Promoting tissue repair and regeneration.	- High tissue penetration capability with precise targeting.<br>- Reduced drug dosage requirements, improving therapeutic efficiency.<br>- Stable propulsion in deep tissues.	Precli nical (anim al model s)	[38], [39]

**Joint cavities**	- Arthroscopic surgery for targeted drug injection (e.g., cartilage injuries).<br>- Intra-articular injection for treating arthritis or joint pain.	- Invasive procedures with potential complications.<br>- Rapid drug clearance from joint cavities.<br>- Limited precision in accessing cartilage fissures.	- Magnetic robots: positioned via external magnetic fields.	- Drug delivery to specific intra-articular regions.<br>- Cartilage repair and tissue regeneration.<br>- Prolonged retention at injury sites for therapy.	- Non-invasive or minimally invasive delivery, reducing post-surgical complications.<br>- Ability to access cartilage fissures, enhancing therapeutic outcomes.<br>- High-precision delivery, minimizing drug waste.	Precli nical (anim al model s)	[36]
**Inner ear**	- Systemic drug delivery (e.g., oral or intravenous).<br>- Intratympanic injection for local delivery.<br>- Cochlear implants for drug administration.	- Blood-labyrinth barrier limits systemic drug delivery.<br>- Poor drug diffusion into cochlear and vestibular regions.<br>- Intratympanic injections are invasive and may cause damage.	- Magnetic robots: guided by external magnetic fields for precise navigation.	- Targeted drug delivery for inner ear diseases (e.g., otitis media).<br>- Regular removal of biofilms on the surface of ear implants.	- Non-invasive or minimally invasive delivery, reducing risks of cochlear damage.<br>- Long-term retention in cochlear and vestibular fluids.	*Ex vivo* studie s	[65]
**Cerebr ospinal fluid system**	- Lumbar puncture for drug delivery or sampling.<br>- Ventricular catheters for localized drug delivery.	- CSF flow limits drug retention.<br>- Limited precision in targeting specific brain regions.<br>- Invasive procedures may cause infections or brain damage.	- Magnetic robots: controlled by external magnetic fields for movement in CSF.	- Targeted drug delivery to specific CSF regions.	- Non-invasive or minimally invasive delivery, reducing infection risks.<br>- Precise navigation and prolonged retention in CSF flow environments.<br>- Improved drug bioavailability in the brain.	Precli nical (anim al model s)	[11]
